# Pasteur and chirality: A story of how serendipity favors the prepared minds

**DOI:** 10.1002/chir.23349

**Published:** 2021-08-06

**Authors:** Ghislaine Vantomme, Jeanne Crassous

**Affiliations:** ^1^ Institute for Complex Molecular Systems and Laboratory of Macromolecular and Organic Chemistry Eindhoven University of Technology Eindhoven The Netherlands; ^2^ Univ Rennes, CNRS, ISCR ‐ UMR 6226 Rennes France

## Abstract

During the XIXth century, France has been the theater of many discoveries in stereochemistry thanks to prestigious physicists, chemists, and crystallographers. Among them, Louis Pasteur is certainly the father of molecular chirality (the so‐called molecular dissymmetry), and because of the exceptionally favored conditions of his famous discovery on the resolution of tartrates, his findings have often been attributed to serendipity. But let us not forget that “in the fields of observation, chance only favors the prepared minds.”

## Graphical abstract

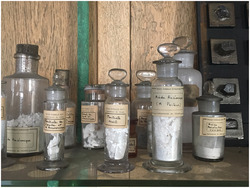

Legend has it that in 1848 Louis Pasteur (1822–1895), young *Agrégé Préparateur* at *Ecole Normale Supérieure* in Paris (Figure 1), was observing crystals of the double sodium‐ammonium salt of tartaric acid under a microscope. To his surprise, he noticed that each crystal has a tiny facet on one of its edges oriented sometimes to the right and sometimes to the left.^1^, ^2^, ^3^ He could separate them by hand with a tweezer. That was the first artificial chiral resolution in the history of science. Since the 1950's and the disaster of thalidomide on infants, chirality has been predominant in the development of biologically active compounds; more recently chirality has enabled the emergence of new nanotechnologies.^4^, ^5^

**FIGURE 1:**
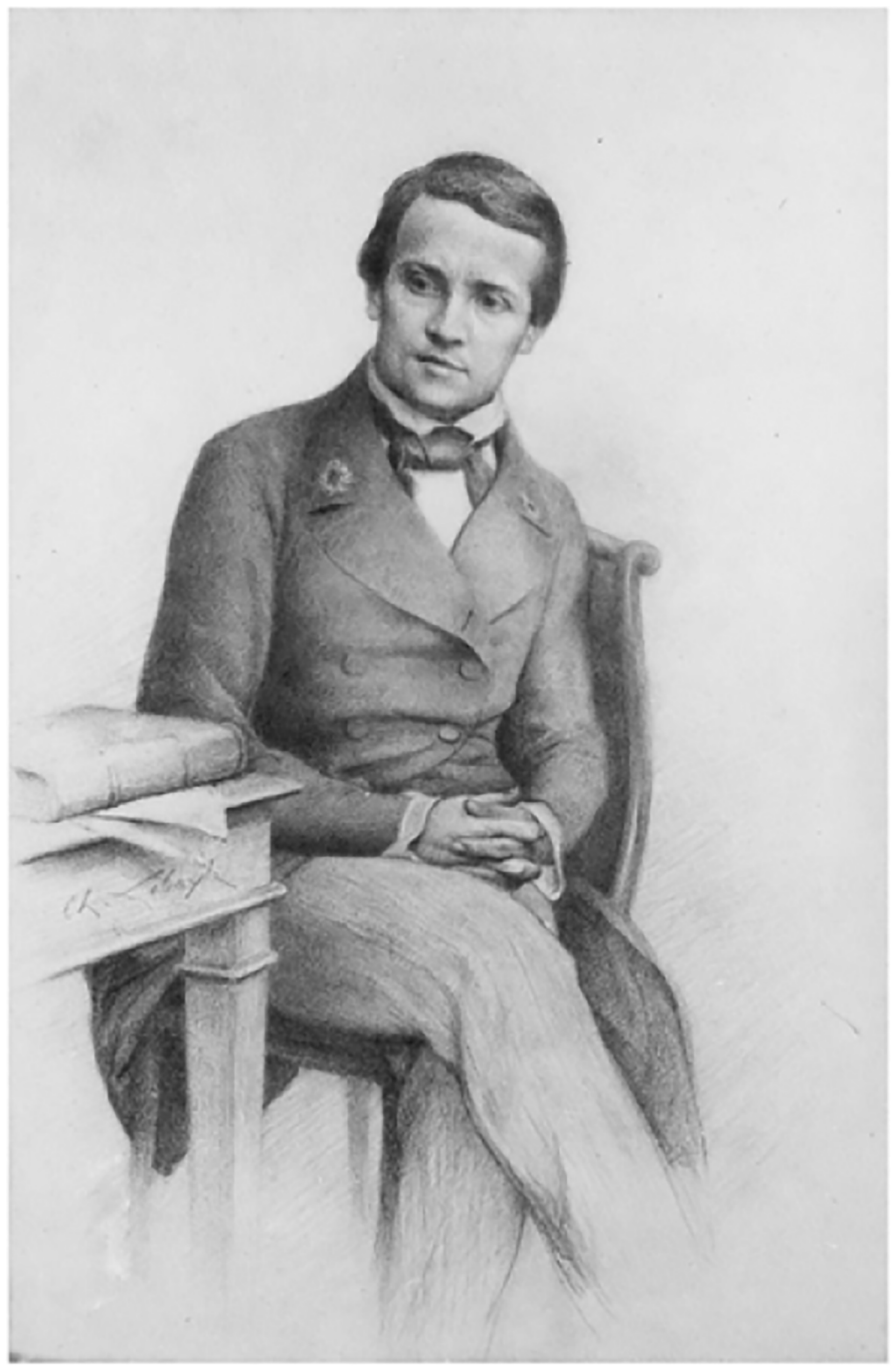
Louis Pasteur at *Ecole Normale Supérieure*. By: Charles Lebayle. Drawing from around 1844. Rights: Coll. musée Pasteur. © Institut Pasteur/Musée Pasteur

Not only the discovery is prominent, but the fact that Pasteur made this finding only 8 months after obtaining his two PhD degrees* on a topic that was thoroughly investigated by many of his contemporary scientists is striking. Looking back at the chain of events that led Pasteur to this discovery can certainly help us learn how scientific discoveries occur and how to favor them in our own laboratories. In this short report, we review the circumstances that brought Pasteur to see what his predecessors had missed before him.

At that time, stereochemistry, that is chemistry in three dimensions,^6^, ^7^ was still not understood as the link between optical activity and molecular structure was missing. The molecular structure of tartaric acid (TA, see Figure 2) was unknown (only its chemical composition was known), and the asymmetric tetrahedral structure of carbon atom was yet to be discovered by Jacobus Henricus van't Hoff (1852–1911) and Joseph Achille Le Bel (1837–1930) in 1874. However, thanks to his famous polarimeter, Jean‐Baptiste Biot (1774–1862) had demonstrated that natural TA and its salts are dextrorotatory (they rotate clockwise the plane of a linearly polarized light) both in aqueous solution and in solid.^8^ From this observation, he deduced that optical activity is linked to individual molecules, and not only to crystal structure. Shortly thereafter, Augustin‐Jean Fresnel (1788–1827) discovered circularly polarized light, and the origin of optical rotation in the difference of refractive indices for left and right linearly polarized light (circular birefringence). Going further, he postulated that the difference between left and right refractive indices should originate from structural effects.^9^ Indeed, Pasteur was aware of the whole literature and of all the breakthroughs accomplished in crystallography and especially on Island Spath crystal and its double refraction properties (observed by Erasmus Bartholinus [1625–1698] in 1669 and utilized by Étienne Louis Malus [1775–1812] in 1808 to define the polarization of light by reflection) and on quartz hemihedral crystals, their molecular arrangement and their ability to deviate the plane of polarized light (seminal works performed by René‐Just Haüy [1743–1822], François Arago [1786–1853], and Jean‐Baptiste Biot between 1801 and 1813).^8^

**FIGURE 2:**
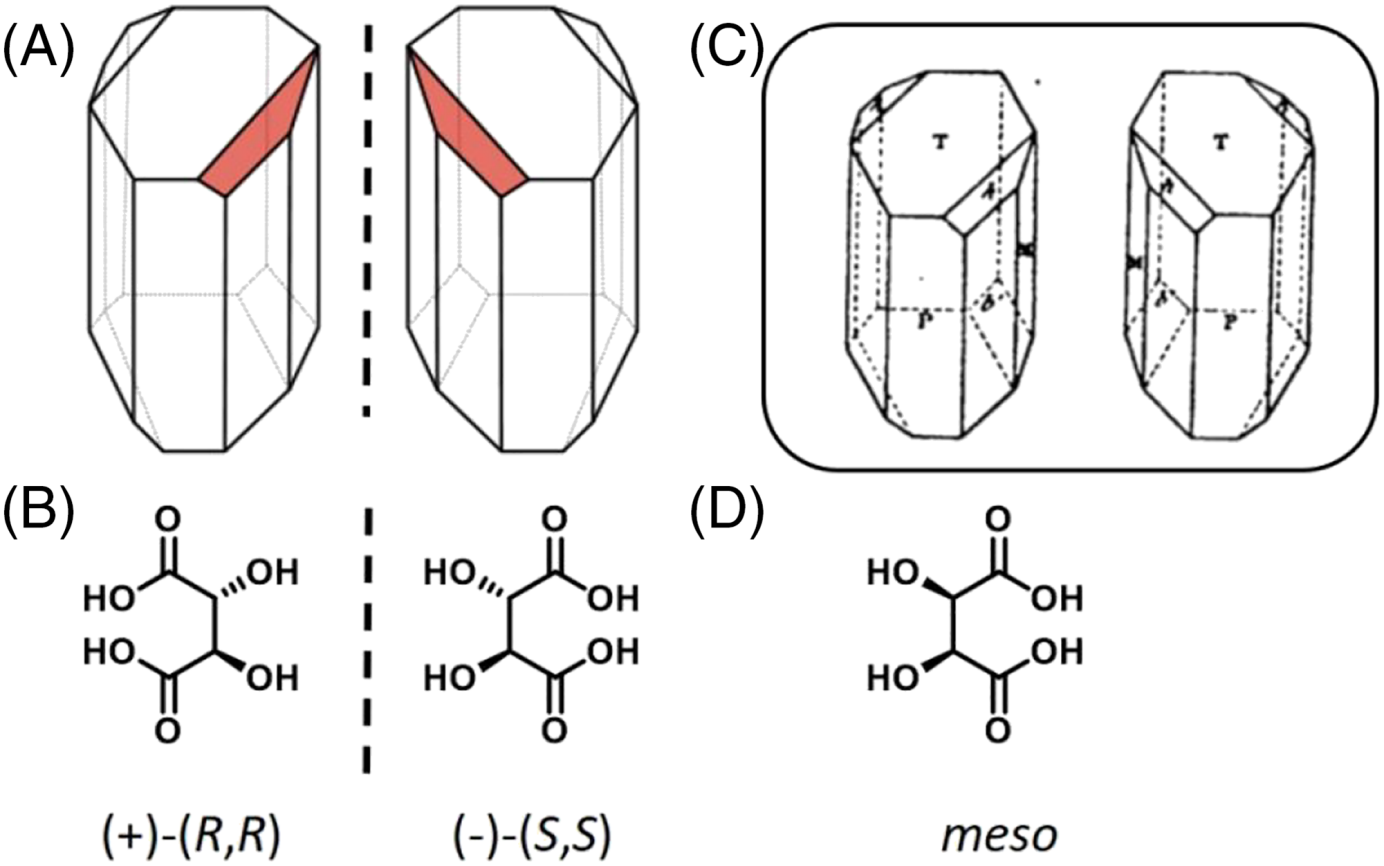
(A) Hemihedral crystals of double sodium‐ammonium tartrates^10^ and (B) chemical structures of enantiomeric tartaric acids. Pasteur describes the facet (in red) that allows to visualize the crystals mirror images. (C) Hemihedral crystals as drawn by Pasteur.^11^ (D) Achiral *meso* stereoisomer of tartaric acid

The story continues when around 1820, a mysterious acid had been unexpectedly prepared by Phillippe Kestner, an industrial chemist located near Strasbourg, from refining TA potassium salt from vinification.^12^ Contrarily to the TA previously studied by Biot, this mysterious acid (the racemic form, later named paratartaric acid by Jöns Jacob Berzelius [1779–1848] and racemic acid by Joseph Louis Gay‐Lussac [1778–1850]) was optically inactive in aqueous and alcohol solutions. Eilhard Mitscherlich (1794–1863), German chemist and crystallographer, undertook to elucidate the chemical composition, crystalline form and physical properties of this paratartaric acid. He studied the double sodium and ammonium tartrate salts and concluded that both crystals, grown from optically active and inactive tartrates,^†^ were identical although they did not interact similarly with linearly polarized light in solution.^13^ It is said that Pasteur did not believe this paradox and, being offered this racemic acid by Kestner,^14^, ^15^ he started the detailed analysis of the crystals. Why do two apparently identical chemicals (double sodium‐ammonium tartrate and paratartrate) have a different effect on polarized light?

The ingenuity of Pasteur was to observe the morphologies of the paratartrate crystals under a magnifying glass. He explained his first hypothesis in a conference in Paris in 1883: “I prepared the double sodium‐ammonium salt of TA and the corresponding paratartrate and I began to compare their crystalline forms, with this preconceived idea that I was going to find the dissymmetry in the form of the tartrate and the absence of dissymmetry in that of the paratartrate. Then, I thought, everything will be explained, […], the dissymmetry of the shape of the tartrate will correspond to its optical dissymmetry; the absence of dissymmetry of shape in the paratartrate will correspond to the inactivity of this salt in terms of the controlled light, to its optical indifference.”^16^ But, to his surprise, he observed that the paratartrate is composed of two types of dissymmetric crystals: “These two tetrahedra are symmetrical (Figure 2A, red facets); they cannot be superimposed: they are in relation to each other what an image is, in a mirror, in relation to the real thing.”^11^

He then continued with his famous experiment: “I carefully separated the right hemihedral crystals and the left hemihedral crystals and separately observed their solutions in the polarization apparatus of Mr. Biot. I then saw, with no less surprise than happiness, that the right hemihedral crystals deviate to the right, that the left hemihedral crystals deviate the plane of polarization to the left, and when I took from each of the two kinds of crystals an equal weight, the mixed solution was neutral for light by neutralization of the two equal and opposite individual deviations.”^11^ Pasteur had demonstrated that the paratartrate is not a pure compound but a 1:1 combination of two enantiomers in a racemic mixture (his tartrate crystals can still be found in Paris, see Figure 3). This important discovery was later reproduced together with Biot who then exclaimed: “Mon cher enfant, j'ai tant aimé les sciences dans ma vie que cela me fait battre le cœur” (“My dear boy, I have loved science so much during my life, that this touches my very heart”).^10^ They proceeded by deduction that the chiral structure of the crystal might come from a dissymmetric structure of the molecule and concluded that molecules with similar chemical and physical properties can have different three‐dimensional organization. The principles of molecular dissymmetry were founded.

**FIGURE 3:**
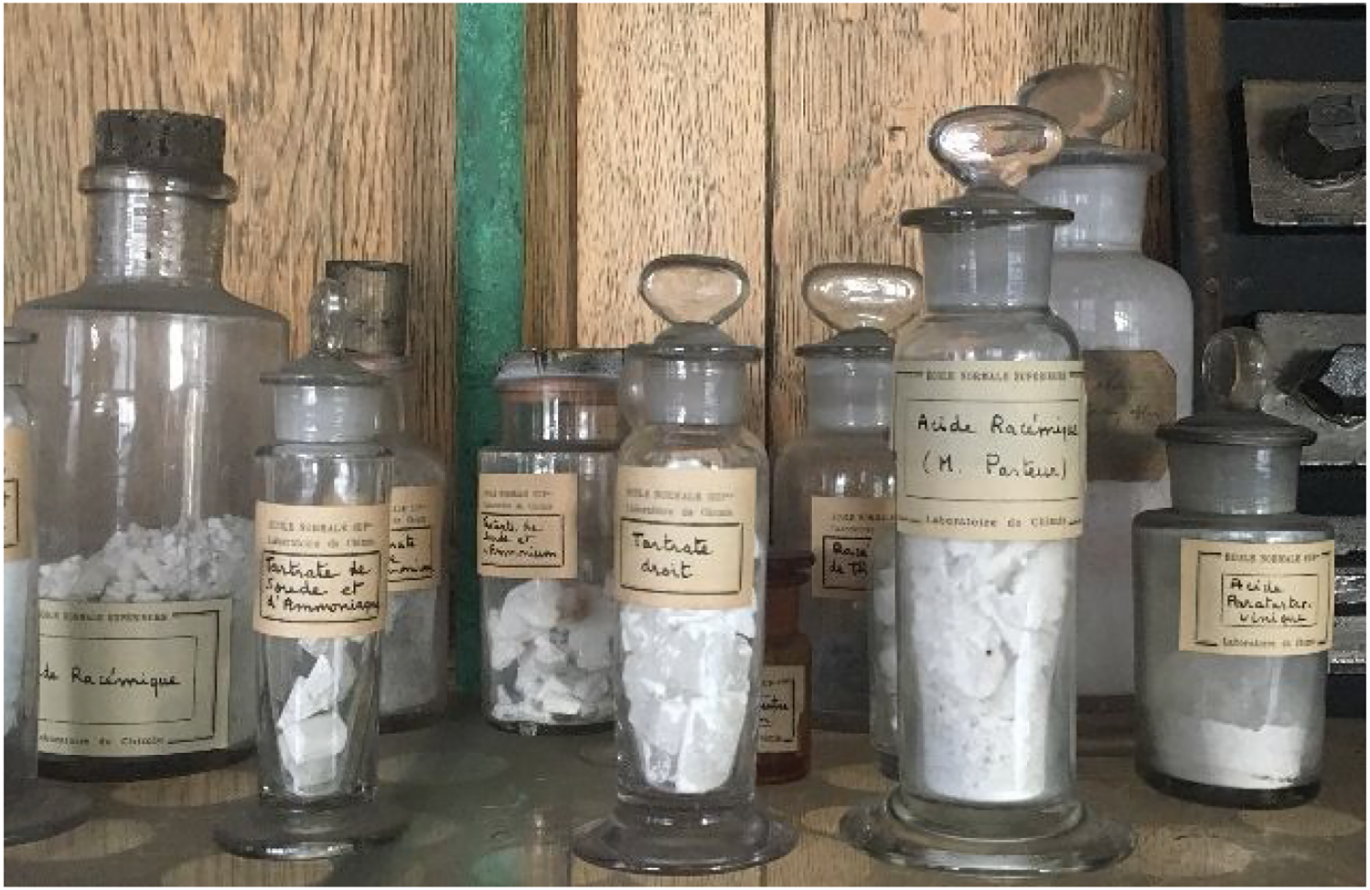
Photograph of Pasteur's crystals in the library of *Ecole Normale Supérieure*, 2019, Paris

Next to his excellent experimental abilities and observation skills, the use of sodium‐ammonium salt of the TA was an exceptionally favorable case for Pasteur.^17^ In fact, less than 10% of racemic species can be resolved spontaneously by crystallization in the form of conglomerates (a physical mixture of two enantiomorphous crystal types, each containing only one of the enantiomers) instead of racemates (crystals each of which contains the two enantiomers in 1:1 ratio).^18^ And the hemihedry and spontaneous resolution are two rare occurrences. Moreover, obtaining this conglomerate is highly dependent on experimental conditions and here again Pasteur's careful observations have been crucial. He rigorously reported that “the crystallization must be undertaken in the morning, because the temperature elevation during the day makes the crystals partly redissolve, which makes the small hemihedral facets disappear.”^19^ Indeed, at 20°C, the sodium‐ammonium tartrate crystallizes as a conglomerate and at 30°C as a racemate due to the dehydration of the conglomerate occurring at 28°C. By chance, he could have missed it! Altogether, it took more than 40 years for the second case of conglomerate to be reported in 1886 by Arnaldo Piutti (1857–1928) with asparagine,^20^ proving the exceptional conditions that led Pasteur to his discovery. Later on, Pasteur continued with his work on dissymmetry and achieved important contributions to early stereochemistry, such as (1) the phenomenon of diastereoisomerism, (2) the resolution of racemic mixtures via fractional crystallization of diastereoisomeric salts, (3) the racemization of the tartaric acid enantiomers and the existence of *meso*‐tartaric acid (“inactive tartaric acid,” see footnote 2), (4) the use of living systems and the fermentation of tartaric acid as an enantiomeric resolution process, (5) the search for the existence of dissymmetric forces in the universe.

Beyond favorable experimental conditions, his findings may have also been influenced by his artistic talents, as recently suggested by Joseph Gal.^21^ Indeed, Pasteur was trained as a lithography artist, which made him familiar with the mirror image inversion. In lithography, the final image on paper is the mirror image of the original drawing on a limestone stone and to improve the quality of his drawings, Pasteur made them by looking at their image in the mirror.

Although built upon fundamental work started by others, the discovery of Pasteur has been called serendipitous, defined in our modern society as “occurring or discovered by chance in a happy or beneficial way.”^22^ However, this definition is reductive and omits a fundamental aspect to the original sense of serendipity as defined by its inventor Horace Walpole: the concept of chance together with discernment.^23^ The modern definition only stresses the importance of accidental discoveries in what then was primarily attributed to the “prepared mind”. Serendipity requires the capacity to work with the unexpected, to take into account the share of creativity in the production of knowledge forged by education and culture, and accumulated over experience and reflexivity.^24^ In other words, *the process of discovery is not a blissful posture but a capacity of being astonished, of being conscious of something unusual, of thinking critically and trying to imagine an interpretation*. But does the modern reduction in the definition of serendipity tell us something about our modern approach to science and discovery? It certainly distorts our vision of Pasteur's achievements and highlights the unrecognized importance of fundamental work and basic knowledge leading to breakthrough discoveries. It reflects on the superiority of applied science and more specifically targeted science (science with very defined objectives) at the expense of basic research in our modern societies, as visible by the decrease in governmental funding for basic research in the last decades.^25^

Discoveries are aspirations arising from curiosity. To create a fertile environment where discoveries happen, researchers should be given time to take risks and fail, space to explore and diverge from their original plans, and means to provide their opportunities. Modern methods such as machine learning may play an important role in performing new breakthroughs in finding new knowledge and new chemical artificial systems.^26^ It also goes in concert with the training of outstanding scientists who are knowledgeable and passionate about their subjects in a multidisciplinary scientific approach. We hypothesized that Pasteur would have agreed with this correction of the current definition of serendipity, as says his famous quote: “Maybe you will say, by chance, but remember that in the fields of observation, chance only favors the prepared minds”.^27^

However, the story is not so simple. Really, Louis Pasteur was a genius in the sense that his numerous researches always started from everyday life observations and from a question of applied interest (an accident in a chemical company, the problem of how to preserve wine during a long journey in a boat, how to control the fermentation process for making alcohol from beetroots, how to treat a patient infected with rabies, etc.) from which he took the opportunity to develop very fundamental science and understand general phenomena that changed the world and are still today of prime importance. Louis Pasteur was a great observer, a highly talented experimentalist, and he had outstanding deduction skills. He importantly deduced from all his observations that molecular chirality may have been at the origin of life, a question still intensively debated. But his discoveries were always derived from an unexpected event, a first observation, a first astonishment. In that sense, throughout his entire career Pasteur dealt with serendipity. In fact, we can reasonably say that serendipity is a process at the heart of any important scientific discovery.^28^

## Data Availability

Data sharing is not applicable to this article as no new data were created or analyzed in this study.

## ENDNOTES

* Pasteur prepared two PhD theses he defended in Paris in 1847, one in chemistry on arseniate derivatives, and one in physics on the optical rotation of liquids.

† Here, the inactive tartrate refers to the racemic mixture (2R,3R)/(2S,3S) and not to the meso form (see Figure 3). The latter achiral form of tartaric acid was also discovered in 1858 by Pasteur upon heating and racemizing the tartrate during his experiments related to the preferential crystallization of diastereoisomeric salts.^29^
